# Visualizing mitochondrial dynamics at the nanoscale

**DOI:** 10.1038/s41377-024-01582-3

**Published:** 2024-09-09

**Authors:** Till Stephan, Peter Ilgen, Stefan Jakobs

**Affiliations:** 1 Buchmann Institute for Molecular Life Sciences, Goethe University Frankfurt am Main, Frankfurt am Main, 60438 Germany; 2 Department of NanoBiophotonics, Max Planck Institute for Multidisciplinary Sciences, 37077 Göttingen, Germany; 3 Clinic of Neurology, University Medical Center Göttingen, Göttingen, 37075 Germany; 4 Fraunhofer Institute for Translational Medicine and Pharmacology, Translational Neuroinflammation and Automated Microscopy, Göttingen, 37075 Germany

**Keywords:** Optics and photonics, Imaging and sensing

## Abstract

The study of mitochondria is a formidable challenge for super-resolution microscopy due to their dynamic nature and complex membrane architecture. In this issue, Ren et al. introduce HBmito Crimson, a fluorogenic and photostable mitochondrial probe for STED microscopy and investigate how mitochondrial dynamics influence the spatial organization of mitochondrial DNA.

Mitochondria have a unique double membrane architecture: surrounded by a smooth outer membrane, the mitochondrial inner membrane (IM) forms invaginations called cristae that protrude into the interior of the organelle, thereby creating defined microcompartments for the molecular machinery of the oxidative phosphorylation (OXPHOS) system. In mitochondria of many cell types, these cristae are densely packed along the mitochondrial tubules at intervals of about 70–100 nm, making them invisible to conventional, diffraction-limited fluorescence microscopy. Thus, electron microscopy of fixed samples has been the primary tool for visualizing cristae for many years. However, the advent of live-cell compatible super-resolution microscopy (SRM) has provided an effective solution for the investigation of mitochondrial dynamics at the nanoscale. With optical resolutions of 100 nm or better, structured illumination microscopy, stochastic optical reconstructions microscopy, and stimulated emission depletion (STED) microscopy have all been used to visualize the IM in live cells^1^. Among these, STED microscopy excels due to its ability to achieve sub-100 nm spatial and second-scale temporal resolution in live samples. However, live-cell STED microscopy requires highly efficient and specialized fluorescent labels.

Only recently, several labeling strategies have been developed for STED imaging of cristae (Fig. 1), including exchangeable pan-membrane labels^2^, chemogenetic approaches^3^ and, notably, IM-specific organic fluorophores^4–8^. The latter in particular have been a focus of development in recent years, resulting in a growing collection of highly advanced IM labeling probes with different properties (Fig. 1). Here, Ren et al., pioneers in developing labeling strategies for SRM, have expanded the palette of mitochondrial probes for STED microscopy with “HBmito Crimson”, a fluorogenic mitochondrial probe combining low saturation power, exceptional brightness and high photostability with excellent biocompatibility^9^.

**Fig. 1:**
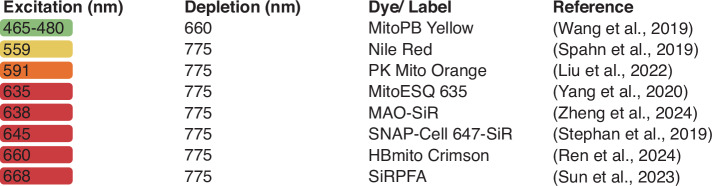
Fluorophores for live-cell STED imaging of cristae

To create this probe, the authors functionalized a silicon rhodamine (SiR) with a lipophilic octanoate alkyl residue. This modification effectively prevents the SiR probe from accumulating in the mitochondrial matrix, and directs it to accumulate in the IM instead. Remarkably, HBmito Crimson exhibits a substantial increase in fluorescence when bound to membranes, enabling high-contrast labeling and wash-free cell recordings. Due to its spectral properties (*λ*_ex_ = 660 nm, *λ*_em_ = 688 nm), HBmito Crimson undergoes efficient depletion by 775 nm STED light with minimal re-excitation. As a result, Ren et al. achieved an excellent resolution increase at STED intensities of only ~35 mW, which is ~10–30% lower than with previously introduced labels^3,4,9^. Using HBmito Crimson, the authors resolved mitochondrial cristae in live mammalian COS-7 cells in 2D and 3D, achieving optical resolutions of up to ~40 nm.

Mitochondria are unique organelles because they carry their own circular genome that is a remnant of their prokaryotic origin. The human mtDNA encodes 13 proteins, 2 ribosomal RNAs, and 22 tRNAs. The mitochondrially encoded proteins are all highly hydrophobic, essential proteins of the OXPHOS system, and mutations in the respective genes are associated with severe disease. A single mtDNA molecule is compacted by proteins into a structure called a nucleoid, of which there are typically several hundred in the aqueous matrix space of the mitochondria of a single cell^10^. The maintenance of mtDNA and the distribution of nucleoids are crucial for cellular homeostasis. Therefore, it has been suggested that mechanisms exist to ensure the proper distribution of mtDNA within the organelle network, even in the midst of continuous mitochondrial tubule fusion and fission events. However, the detailed molecular nature of these mechanisms, which ensure the proper positioning of nucleoids within the mitochondria, is still not clearly defined.

In their study, Ren et al. used HBmito Crimson in combination with the fluorescent DNA intercalator dye SYBR Gold to highlight cristae and the nucleoids. Nicely aligning with other reports^3,4,9^, they found that nucleoids are generally situated in voids between mitochondrial cristae stacks. Thereby, the nucleoids show a regular distribution across the mitochondrial networks, with a preference for localization at tips and branch points. They demonstrate that at these branch points, the cristae adopt a specific arrangement, resulting in a matrix-filled void at the center of the branch point, which is occupied by a nucleoid in about two-thirds of cases. Interestingly, the authors’ high-resolution time-lapse recordings show that dynamic IM remodeling influences nucleoid position and separation, demonstrating that mitochondrial fusion and subsequent cristae remodeling can mediate the transfer of nucleoids from previously separated mitochondria. All these observations suggest that cristae may act as barriers within the matrix space, thereby defining the localization of nucleoids, but also promoting sub-compartmentalization of the mitochondrial matrix (Fig. 2). Indeed, it has previously been shown that MICOS mutants lacking lamellar cristae no longer have well-spaced nucleoids, but the nucleoids tend to aggregate^4^.

**Fig. 2:**
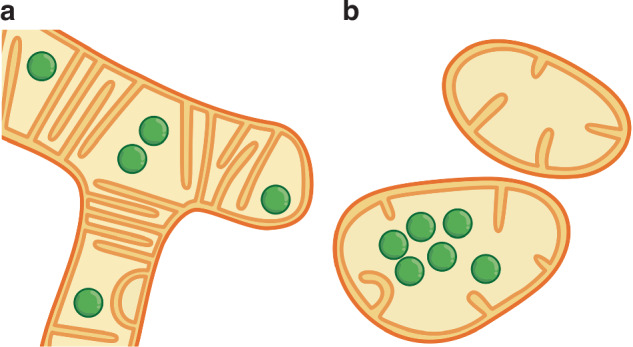
**Illustration of mitochondria and nucleoids under physiological conditions and upon stress-induced mitochondrial remodeling.** **a** In healthy mammalian cells, mitochondria typically form extended, branched networks that are filled with densely stacked cristae. Voids between stacks of cristae are often occupied by nucleoids (green) that are regularly distributed throughout the mitochondrial network. **b** Induced by cellular stress, loss of cristae shaping proteins or the induction of cell death, the mitochondrial network fragments and the mitochondrial ultrastructure is disturbed. As shown by Ren et al.^9^, this process is accompanied by an aggregation and disorganization of the nucleoids, suggesting that cristae are critical to compartmentalize the matrix space

Intriguingly, the authors report that during cell death such as apoptosis or ferroptosis, the cristae architecture is disrupted with consequences for the sub-organellar distribution of nucleoids. Following the induction of cell death, mitochondrial networks fragment, individual mitochondria become rounded up, and the overall cristae structure is disrupted. These morphological changes coincide with the accumulation of nucleoids in the center of the fragmented mitochondria (Fig. 2). These observations support the idea that not only do nucleoids, as they encode for OXPHOS proteins, influence the cristae architecture, but that also the cristae architecture influences the localization of the nucleoids. When cristae are lost, nucleoids aggregate, presumably driven by phase separation processes.

In recent years, several studies using live-cell SRM have described mitochondrial cristae dynamics^3,11,12^. Although the functional implications of these movements are not yet fully understood, it is becoming clear that changes in IM dynamics are linked to alterations in cristae architecture, but also influence the overall mitochondrial network structure. This article^9^ presents new evidence that these nanoscale IM dynamics are also key to the distribution of the gene expression machinery within the aqueous matrix space.

Ren et al. highlight the need for live-cell imaging at nanoscale resolution to understand the complex biology of mitochondria. We predict that next-generation fluorophores, such as HBmito Crimson, will be invaluable tools for elucidating the mechanisms by which mitochondria control their dynamics and how these changes relate to the organelle’s various functions in health and disease.
